# Measuring intra-individual physical activity variability using consumer-grade activity devices

**DOI:** 10.3389/fdgth.2023.1239759

**Published:** 2023-09-06

**Authors:** Vered Lev, Marily A. Oppezzo

**Affiliations:** ^1^ Stanford Prevention Research Center, Stanford University School of Medicine, Stanford, CA, United States; ^2^ Department of Medicine, Stanford Prevention Research Center, Stanford University, Stanford, CA, United States

**Keywords:** behavior change, sedentary behaviors, physical activity intervention, activity trackers, intra-individual, wearables

## Abstract

Many existing sedentary behavior and physical activity studies focus on primary outcomes that assess change by comparing participants' activity from baseline to post-intervention. With the widespread availability of consumer-grade devices that track activity daily, researchers do not need to rely on those endpoint measurements alone. Using activity trackers, researchers can collect remote data about the process of behavior change and future maintenance of the change by measuring participants’ intra-individual physical activity variability. Measuring intra-individual physical activity variability can enable researchers to create tailored and dynamic interventions that account for different physical activity behavior change trajectories, and by that, improve participants' program adherence, enhance intervention design and management, and advance interventions measurements' reliability. We propose an application of intra-individual physical activity variability as a measurement and provide three use cases within interventions. Intra-individual physical activity variability can be used: prior to the intervention period, where relationships between participants' intra-individual physical activity variability and individual characteristics can be used to predict adherence and subsequently tailor interventions; during the intervention period, to assess progress and subsequently boost interventions; and after the intervention, to obtain a reliable representation of the change in primary outcome.

## Introduction

A large body of evidence demonstrates that regular physical activity (PA) contributes to a range of physical and mental health benefits (1, 2). High amounts of sedentary behaviors (≥8 h/d) have been associated with increased risks of various negative health outcomes including incident cardiovascular disease, type 2 diabetes and all-cause mortality (3). Increasing physical activity is a behavior change implemented in both sedentary behavior and physical activity studies. Individuals that increased their physical activity showed improved health and well-being outcomes as reductions in psychological distress, improved perceived quality of life, and decreased systolic BP (4, 5). To date, much of this evidence base regarding physical activity behaviors have come from studies where the primary outcome is assessed by comparing participants' measures of activity from 7 day of monitoring at baseline to post-intervention measures. With the widespread availability of consumer-grade activity tracking devices, research no longer has to rely on endpoint measurements alone. In fact, key insights about the process of behavior change and future maintenance of the change may lie in the days between.

One way to look at the daily process data is through means- e.g., mean steps per day. However, variability around the mean is a better description of the actual process through which a person's mean steps may change throughout an intervention. Intra-individual variability has been used in different health and medical areas as a predictor for health outcomes. However, intra-individual physical activity is a relatively underutilized variable for physical activity interventions. A recent Technology and Healthcare paper posed physical activity variability as an important and relatively underutilized measure both in physical activity interventions and clinically in evaluating patient health (6). A PubMed search for the last 5 years using search terms (“physical activity variability”), (“intra-individual variation” and “physical activity”), and (“physical activity” and “intra-individual variability”) yielded 6 results which actually used the variability of physical activity as the primary outcome.

Many studies have been using heart rate variability to measure different health outcomes using wearables. For example, researchers were able to predict general health and mental health measures using heart rate variability data collected from wrist wearables (7). Wearable-measured resting heart rate variability during sleep was a predictor of perceived physical fitness on the subsequent morning (8). Variability also gained popularity in other health areas, for example, high HbA1c variability was found to be associated with increased risk of diabetic complications, all-cause and cardiovascular mortality (9). As intra-individual variability measures are showing substantial value in those areas, we hope to encourage physical activity and behavior change researchers to further adopt this measurement. Intra-individual physical activity variability is the within-person standard deviation from the person's average physical activity over a predetermined period of time. As discussed in more details later in the paper, intra-individual coefficient of variation can be calculated in order to show the extent of variability in relation to the mean. Researchers can evaluate participants' intra-individual physical activity variability on a day-to-day level (e.g., comparing average daily steps in day 1 to day 2) or on a weekly level (e.g., comparing the average daily steps on week 1 to the average daily steps on week 2). Physical activity variability can be used in sedentary behavior and physical activity interventions to 1. improve participants' program adherence; 2. enhance intervention design and management; 3. advance interventions measurements' reliability. Each is described in detail below.

### Improve program adherence and enhance intervention design and management

To improve program adherence and enhance intervention design, researchers could look for factors that might predict individuals' behavior change trajectories. It is feasible that some personal characteristics correlate with intra-individual physical activity variability patterns and could indicate future behavior change trajectory. For example, it is possible that baseline readiness to change levels, specificity of goals, or tolerance for flexibility may correlate with certain variability patterns and change throughout the intervention. Similarly, in one study, Watts et al. (10) found that men have a significantly higher physical activity variability than women (in an older adults' sample). Identifying these potential predictive relationships could help create a more personalized intervention design that match individuals' different variability patterns and personal characteristics to maximize their chances to adhere to the PA program.

### Assess physical activity measurements' reliability

Measuring intra-individual PA variability could also help researchers assess physical activity measurements' reliability. Evidence in the literature supports the claim that when assessing change, the degree of variability should be considered for reliable representation of change, especially when using single time period (e.g., last week of the study). Salthouse & Nesselroade research on short-term fluctuations and Rowlands et al.'s study on PA variability identified the issue that *the larger the “noise” associated with short-term fluctuation, relative to the “signal” that corresponds with real change, the harder it is to identify the real change*, meaning that the magnitude of an individual's short-term variations in physical activity can interfere with the assessment of accurate change in activity (11, 12). In addition, restricting measurement of an activity outcome to just a short pre-intervention and post-intervention measurement period risks reliability of being a marker of a person's “true” behavior and the person's historical intra-individual variability can provide useful data about the relative reliability of their endpoint measurement.

## Considerations

When aiming to utilize intra-individual physical activity variability, one should consider the frequency in which the variability would be measured. Assessing PA variability on the daily level could be challenging due to the normal variability in daily life and circumstances that affect PA as weather, mood, or schedule. In addition, people have different physical activity routines that might not be well represented on a day-to-day level (for example, some people might be very active any other day and not every day). However, when measuring intra-individual variability on the weekly level (over a 7-day period), daily fluctuations and different physical activity schedules and routines are factored in. A recent study evaluated the number of observation days required to provide reliable estimates of participants' habitual activity and found that protocols that result in 7–10 valid observation days for each participant may be needed to obtain reliable measurements of key PA measures (13).

Intra-individual weekly variability during an intervention period can be estimated using consumer-grade activity devices. For example, using each participant's daily step data, weekly averages of the daily steps can be calculated for each week of the intervention. Then, to indicate the degree of weekly physical activity variability for each participant, an intra-individual coefficient of variation (CV) can be calculated. CV is the ratio of the standard deviation to the mean, showing what proportion of the mean the person's variability is. The ratio standardizes the value to correct for total steps; otherwise, a person with a low mean might have a lower variability due to a truncated range. CV is calculated for each participant from the mean of their daily step average - for each of the intervention weeks - and their standard deviation (SD). The higher the CV, the higher the daily fluctuation in activity–which could be indicative of absence of routine and consistency. With that, variability is n't necessary bad. If a participant has a high variability (CV) around their daily steps average, and their average for the week is close to the target steps, it could indicate that in some days they make the targeted daily steps, and some days they fall short. However, if a participant has a high variability around a mean that is quite far away from the target, this may reflect inadequate effort to change and reach their target steps.

### Limitations of wearable activity trackers in practice

It should be noted that while wearable activity trackers enable a better measurement of the dynamic behavior change processes during PA interventions, there are limitations. Some of these include inaccurate measurements for some activities such as weightlifting and swimming, loss of data due to synchronization errors, third-party data collection companies with unexpected algorithm changes, and participants' behaviors changing due to simply wearing an activity tracker that provides user feedback (14).

## PA intra-individual variability within interventions - relevant intervention phases

### Pre-intervention: predicting individual's behavior change

Chrzanowski-Smith et al. (15) encouraged sport and exercise scientists to acknowledge intra-individual variation in baseline and prior to the implementation of an intervention and suggested that pre-intervention intra-individual PA differences can offer insights that may predict or explain individual responses to an exercise program (15). One example Chrzanowski-Smith et al. mentioned regarding the benefits of intra-individual variation measurement in baseline physical activity is the potential impact that intra-individual variation may have for stratified randomization. They proposed that by identifying intra-individual variation prior to intervention randomization we could allow a more suitable participants' matching and intervention groups' assignments as those baseline training characteristics enable a more accurate representation of the participants' activity levels and practices.

For multi-phase and pilot interventions, researchers can use their first round of data collection to identify relationships between baseline individual's characteristics and individual's physical activity variability patterns throughout the intervention in order to design future interventions that help account for those variability patterns. For example, consider an individual who starts a daily walking program with a low tolerance for flexibility or an all or nothing mentality. A possible variability pattern that could show up during the intervention is that as soon as that person deviates from their target (high variability) they return back to baseline for a week. If there were strong correlations between low tolerance for flexibility or all or nothing mentality and this pattern of variability, one could design an intervention with a booster session partway through the intervention month to help the person overcome these behavior patterns.

### During-intervention: identifying participants' dynamic and unique behavior change

Rowlands et al. (12) noted that knowing a person's degree of variability in physical activity may facilitate a more attentive and personalized approach to interventions. Measuring PA variability during an intervention can enable researchers to create interventions that gradually work in tandem with participants' PA variability and behavior (change) and suit the needs of the person in order to maximize adherence to the PA program and success. Intra-individual variability could provide researchers with real-time feedback for program adherence and help us better understand the timeframes in which participants are able to sustain a new or intended behavior. However, this type of variability would be more of a “success variability”, meaning it is the daily/weekly distance participants' are from their daily/weekly target. For example, if an individual set a daily goal of 10,000 steps a day, success variability would illustrate the daily deviation from that goal. In a day where the participant walks 7,000 steps, the success variability would be negative 3,000 steps. Researchers can choose time points during the intervention where they assess participants' deviation range around their intended mean and modify participants' interventions accordingly. Assessing participants' deviation range around their intended mean would allow researchers to decide what would a normal deviation range from the intended mean that would still allow program adherence and either consider finding a more appropriate daily/weekly goal for those who fall outside the range or introduce a theory-based behavior change technique to potentially help participants achieve lower variability around their intended mean. Lastly if a participant had shown “positive success variability” meaning, they demonstrated higher daily/weekly mean then they intended, (e.g., they walked an average of 12,000 steps a day instead of 10,000) then researchers can also work with them to find a more appropriate goal. Theses insights could help researchers facilitate individuals' dynamic behavior change and inform more tailored physical activity interventions, programs and guidelines that account for individuals' unique behavior change and support individuals' differences and barriers to achieve PA goals.

### Post – intervention: determining change outcomes

In some cases, the richness of the data about the actual process through the intervention could change the interpretation of an individual's change outcome. For example, consider an intervention that measures participants' physical activity levels for one month. Two participants had an identical baseline PA level. Participant A had a very active week at the beginning of the intervention, and they walked 38 miles that week. However, Participant A spent the rest of the month mostly sedentary and walked an average of 4 miles each week for the rest of the intervention month. Participant B walked an average of 14 miles each week of the intervention. In this case, participant A walked a total of 50 miles during the month and participant B walked a total of 56 miles during the month. Even though there is only a slight difference in *total* activity between the two participants, participant A's physical activity is highly variable and participant B has a much more stable physical activity variation. Their physical activity trajectory is very different and might result in different physical activity and future behavior change maintenance outcomes. Solely measuring their change in activity from baseline wouldn't show these critical insights.

Intra-individual variability could also be used during the post-intervention measurement period to help present a more reliable representation of the results. For example, during the post-intervention measurement week, participant A had high intra-individual physical activity variability. On the first two days they walked about 10,000 steps, the following two days they walked 3,000 steps and on the last three days they walked 5,000 steps. With this high intra-individual PA variability, it can be hard to detect the true representation of daily steps at that week and an accurate estimate of the daily step change from baseline. However, participant B had low intra-individual physical activity variability and walked about 7,000 steps almost every day that week. Participant B daily step trajectory makes it easier to determine the real daily step change from baseline with higher certainty.

## Conclusion

With ubiquitous consumer-grade physical activity monitors, physical activity and sedentary behaviors research can go beyond pre-post mean measurement boundaries and analyze participants' dynamic physical activity behavior variability. Measuring intra-individual PA variability could be used to improve participants' program adherence, enhance intervention design and advance measurements' reliability. Intra-individual physical activity variability can be used: prior to the intervention period, where relationships between participants' intra-individual physical activity variability and individual characteristics can be used to predict adherence and subsequently tailor interventions; during the intervention period, to assess progress and subsequently boost interventions; and after the intervention, to obtain a reliable representation of the change in primary outcome. When assessing intra-individual physical activity variability, it might be more appropriate to measure variability on the weekly level rather than on the daily level in order to count for individuals' physical activity schedules, routines, and normal daily fluctuations. We can use consumer-grade activity devices' data to standardize intra-individual physical activity variability across participants by computing coefficients of variation. While current devices still have limitations, the within-subject consistency is adequate, and innovations continue to be made to improve device sensitivity.

## Data Availability

The original contributions presented in the study are included in the article/Supplementary Material, further inquiries can be directed to the corresponding author.
